# Comparative genomics reveals evidence of marine adaptation in *Salinispora *species

**DOI:** 10.1186/1471-2164-13-86

**Published:** 2012-03-08

**Authors:** Kevin Penn, Paul R Jensen

**Affiliations:** 1 Center for Marine Biotechnology and Biomedicine, Scripps Institution of Oceanography, University of California San Diego, La Jolla, CA, 92093-0204, USA

## Abstract

**Background:**

Actinobacteria represent a consistent component of most marine bacterial communities yet little is known about the mechanisms by which these Gram-positive bacteria adapt to life in the marine environment. Here we employed a phylogenomic approach to identify marine adaptation genes in marine Actinobacteria. The focus was on the obligate marine actinomycete genus *Salinispora *and the identification of marine adaptation genes that have been acquired from other marine bacteria.

**Results:**

Functional annotation, comparative genomics, and evidence of a shared evolutionary history with bacteria from hyperosmotic environments were used to identify a pool of more than 50 marine adaptation genes. An Actinobacterial species tree was used to infer the likelihood of gene gain or loss in accounting for the distribution of each gene. Acquired marine adaptation genes were associated with electron transport, sodium and ABC transporters, and channels and pores. In addition, the loss of a mechanosensitive channel gene appears to have played a major role in the inability of *Salinispora *strains to grow following transfer to low osmotic strength media.

**Conclusions:**

The marine Actinobacteria for which genome sequences are available are broadly distributed throughout the Actinobacterial phylogenetic tree and closely related to non-marine forms suggesting they have been independently introduced relatively recently into the marine environment. It appears that the acquisition of transporters in *Salinispora *spp. represents a major marine adaptation while gene loss is proposed to play a role in the inability of this genus to survive outside of the marine environment. This study reveals fundamental differences between marine adaptations in Gram-positive and Gram-negative bacteria and no common genetic basis for marine adaptation among the Actinobacteria analyzed.

## Background

Microbiologists have long sought to define the physiological characteristics of marine bacteria [1]. These studies have largely focused on seawater-inhabiting Gram-negative bacteria. None-the-less, Gram-positive bacteria are consistently reported from marine samples [2]. Among these, representatives of the phylum Actinobacteria are particularly well represented [3,4]. To date, the genetic basis for marine adaptation in the Actinobacteria remains uncharacterized.

Early attempts to define marine bacteria centered on the observation that some marine-derived strains failed to grow when seawater was replaced with deionized (DI) water in the growth medium [1]. Subsequently, this physiological response was linked to a specific sodium ion requirement, which led to the realization that seawater was not simply required for osmotic balance [5]. Based on this, marine bacteria were further defined by a demonstrable requirement of sodium for growth [1]. This requirement was subsequently linked to electron transport [5] and the possession of the sodium pumping respiratory NADH dehydrogenase Nqr (sodium quinone reductase) [6]. In addition to electron transport, it has also been reported that sodium is required for amino acid transporters and for the oxidation of compounds such as alanine and galactose in some marine bacteria [5]. The ionic requirements of marine bacteria can also include calcium and magnesium [1], but the genetic basis for these requirements is unknown. At present, it remains unclear if similar marine adaptations occur in Gram-positive taxa.

The discovery of the sodium-pumping NADH dehydrogenase Nqr [6] and the associated genes *nqrA-F *[7] represented the first genetic link to sodium dependence in Gram-negative marine bacteria. Nqr is one of three types of respiratory NADH dehydrogenases and is known to occur in many Gram-negative marine bacteria and some clinical pathogens [6,8]. When present, Nqr does not preclude the occurrence of other NADH dehydrogenases in a genome [8]. The more common prokaryotic NADH dehydrogenase is the proton-pumping NDH-1, which is also known as complex I [9]. NDH-1 is composed of 14 genes (*nuoA-N*) and displays no homology with Nqr yet both are energy-coupling enzyme complexes that create an ionic motive force used to generate ATP and drive other cellular processes [10]. Interestingly, the membrane-bound, ion pumping *nuo *genes display significant sequence similarity to the six genes that make up the multi-subunit Na^+^/H^+ ^antiporter Mrp (*mrpA-G*) [11]. The third type of NADH deydrogenase is NDH-2, which is typically composed of one to a few proteins [10] and is not an energy-coupling complex or been linked to marine adaptation.

The ability of bacteria to adapt to external changes in the osmotic environment is fundamental to survival [12]. Osmoadaptation in bacteria typically involves the intracellular accumulation of compatible solutes such as glycine and betaine. These compounds are acquired either by de novo biosynthesis or directly from the environment. Bacteria also have mechanisms to survive osmotic down-shock that usually involve a combination of specific (secondary transport) and non-specific (stretch-activated channel) mechanisms of solute efflux together with aquaporin-mediated water efflux [12]. One important mechanism of solute efflux is mediated by the mechanosensitive channel of large conductance (MscL). This membrane bound, stretch-activated channel is common in bacteria and believed to act as an emergency value to release turgor pressure following sudden osmotic downshock [13]. In the marine halophile *Vibrio alginolyticus*, the introduction of *mscL *alleviated cell lysis following osmotic downshock [14] and thus the product of this gene may represent an important mechanism to survive the transition from marine to freshwater environments.

In addition to specific ionic requirements and mechanisms to survive osmotic stress, comparative genomics has been used to identify other mechanisms of marine adaptation. For example, ABC branched chain amino acid (BCAA) transporters are enriched in *Bacillus *spp. adapted to alkaline and marine environments [15]. Once taken into the cell, BCAAs are converted into L-glutamate, which would help acidify an otherwise basic cytoplasm [16]. More recently, an abundance of BCAA transporters was observed in several marine *Roseobacter *strains [17]. BCAA transporters also represent a significant portion of the genes observed in marine metagenomes [18] and thus appear to represent an important marine adaptation. Marine adaptation genes were also identified in the marine cyanobacterium *Synechoccocus*, which has a greater capacity to transport Na^+ ^than freshwater species [19].

Actinomycetes belonging to the genus *Salinispora *occur broadly in tropical and sub-tropical marine sediments [20]. To date, two species (*S. tropica *and *S. arenicola*) have been formally described while a third ("*S. pacifica*") has been proposed [21]. This taxon was described as the first obligate marine actinomycete genus based on a failure to grow when seawater was replaced with DI water in a complex growth medium [22]. It was recently demonstrated that *Salinispora *spp. are capable of growth with as little as 5 mM Na^+ ^if the appropriate osmotic environment is provided [23]. However, it was also demonstrated that cells lyse in low osmotic strength media [24] suggesting a high level of marine adaptation.

The genome sequences of *S. tropica *strain CNB-440 and *S. arenicola *strain CNS-205 along with four unrelated marine Actinobacteria (*Aeromicrobium marinum*, *Janibacter *sp., 'marine actinobacterium PHSC20C1', and *Rhodococcus erythropolis *PR4) and a large number of non-marine strains provided an opportunity to use comparative genomics to identify genes associated with marine adaptation. An earlier comparison of the two *Salinispora *genomes revealed a large paralogous family of genes encoding polymorphic membrane proteins (Pmps) [25]. Although functionally uncharacterized, Pmps appear to be type V autotransporters. The large number of copies observed in the two genomes led to the proposal that they represent an adaptation to life in low nutrient environments and that they form pores that render *Salinispora *spp. susceptible to lysis in low osmotic conditions [25]. The present study expands on that initial observation by employing a phylogenomic approach targeting gene gain and loss events to identify additional marine adaptation genes (MAGs). These analyses reveal that the mechanisms of marine adaptation in *Salinispora *spp. are fundamentally different from those reported for Gram-negative bacteria and that there is no common genetic basis for marine adaptation among the Actinobacteria for which genome sequences are currently available. In addition, the results provide strong evidence that gene loss plays a critical role in the inability of *Salinispora *spp. to survive when seawater replaces DI water in complex growth media.

## Results

### Marine adaptation genes

Two fundamental approaches were used to identify genes associated with marine adaptation in the marine actinomycete genus *Salinispora*. The function-based approach relied on BLAST analyses using key words derived from previously reported marine adaptation genes (MAGs). The comparative genomics approach was annotation independent and detected genes that were present in *Salinispora *species but absent or rare in other Actinobacteria. Thus, the first approach tested for common mechanisms of marine adaptation among marine bacteria while the later had the potential to detect new or unknown gene functions that may be relevant to marine adaptation. All genes detected using these two approaches were then tested for evidence of a recent common ancestry with bacteria associated with hyperosmotic environments.

The function-based approach yielded the largest number of candidate marine adaptation genes (MAGs), however the vast majority identified using both approaches did not pass the phylogenetic test and therefore did not advance to the final MAG pool (Table 1). Ultimately, 60 and 58 MAGs were identified in the *S. tropica *and *S. arenicola *genomes, respectively. Of the MAGs identified based on gene function, 13 are involved in electron transport, 12 encode transporters, and 28-30 (depending upon species) encode channels or pores. Based on comparative genomics, more genes related to marine adaptation appear to have been gained than lost from the two *Salinispora *spp. (Table 1).

**Table 1:** Marine adaptation genes

Species	MAG status	Functional class				Subtotal	Comparative genomics		Total
		
		ETS (complex I)	Na^+ ^transport	ABC transport	Channels and pores		Gene gain	Gene loss	
*S. tropica*	Candidate	35	18	225	35	313	105	4	422
*S. tropica*	Final	13 (2 operons)	3	9 (2 operons)	30	55	7	1*	60**
*S. arenicola*	Candidate	35	19	225	33	312	105	4	421
*S. arenicola*	Final	13 (2 operons)	3	9 (2 operons)	28	53	7	1*	58**

MAGs identified based on functional class and comparative genomics.

*Based on annotation.

**Total is not additive because 3 of 7 genes in the gene gain category were also found in the finvtional analyses.

ETS: electron transport system.

### Species tree

**Figure 1:**
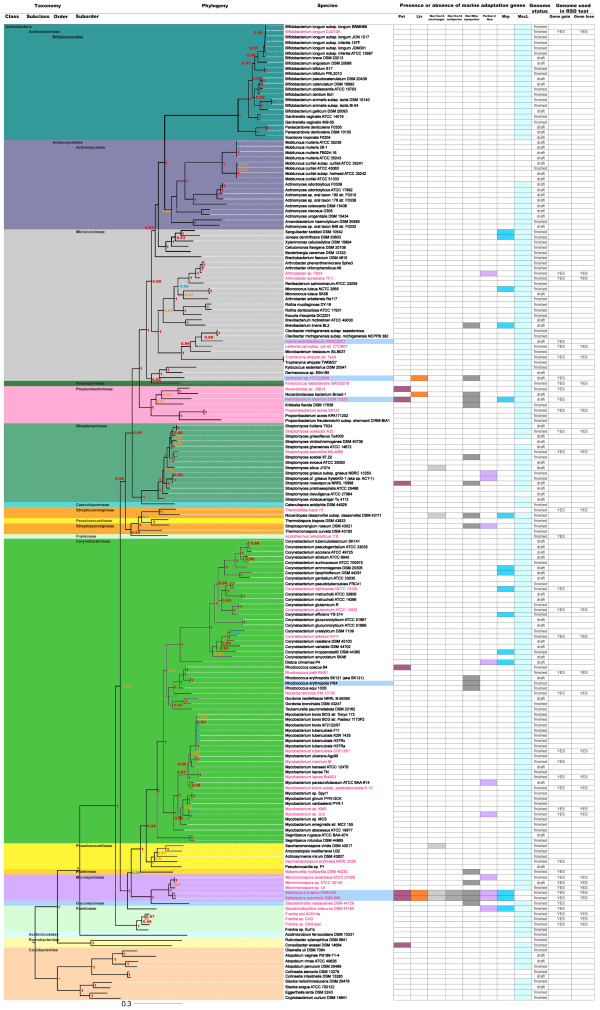
**Actinobacterial species tree showing the distribution of marine adaptation genes**. The Actinobacteria are color coded according to major taxonomic affiliations. Species names are listed vertically and marine adaptation genes (MAGs) listed horizontally across the top of the table. Colored boxes indicate the distribution of each MAG. The names of the 38 strains used in the comparative genomic analyses are colored in pink while the last two columns indicate the genome sequences used for the gene gain and loss analyses. Strains highlighted in blue are of marine origin. Branch support is listed on each node; red values indicate a likelihood of 90 or higher, orange indicates values between 60 and 89 while blue indicates support values below 60. The Coriobacteridae were chosen as the root. Pst, Liv, Partial 2, and Mrp represent all genes in the respective operons. See Additional file 4 for detailed tree parameters.

An Actinobacterial species tree was constructed using 19 of 31 AMPHORA marker genes [26] (Additional file 1) derived from 186 Actinobacterial genome sequences (Figure 1). This phylogeny is largely congruent to that previously published [27] with the notable exception of the close relationship of *Stackebrandtia nassauensis *DSM 4478 (family Glycomycetaceae) to the Micromonosporaceae. This relationship is supported by all of the individual gene trees and has also been reported by others [28]. The tree clearly shows that the marine Actinobacteria for which genome sequences are available are polyphyletic and not deeply rooted. It is also notable that the order Actinomycetales is paraphyletic with respect to the Bifidobacteriales and that the previously reported polyphyly of the families Frankineae and Streptosporangineae is maintained in this tree [29].

### Function-based identification of MAGs

Genes associated with the sodium-dependent NADH dehydrogenase (Nqr), which have been reported in Gram-negative marine bacteria, were not detected in either *Salinispora *genome or in any available Gram-positive marine bacterial genomes. Thus, when it comes to respiratory electron transport, there appear to be fundamentally different mechanisms by which Gram-negative and Gram-positive bacteria have adapted to the marine environment. None-the-less, 35 candidate MAGs with annotation linked to NDH-1 were initially detected in both *Salinispora *genomes (Table 1). These genes comprise three partial and one complete NDH-1 operon (Additional file 2). The 14 genes in the complete NDH-1 operon (*nuoA*-*N*) as well as those in the first partial NDH-1 operon were not considered further because their phylogenies are in general agreement with the Actinobacterial species tree, and thus there was no evidence they had been acquired from marine bacteria.

**Figure 2:**
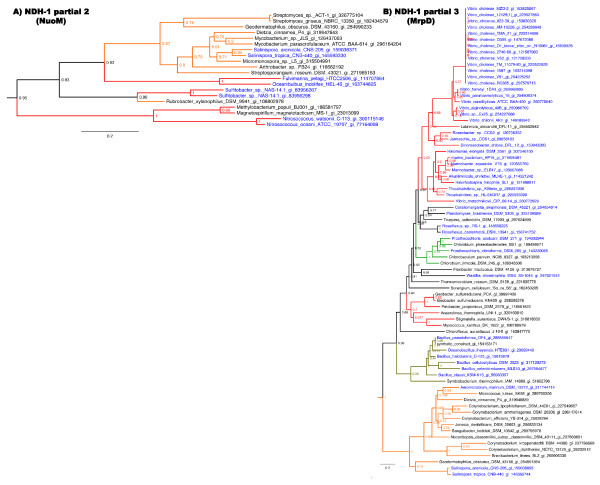
**NADH dehydrogenase-related gene phylogenies**. Representative phylogenies for (A) the NDH-1 partial 2 operon (NuoM) and (B) the NDH-1 partial 3 operon (MrpD). Branch colors: orange = Actinobacteria, red = Proteobacteria, brown = Firmicutes, green = Chlorbi, Pink = Cyanobacteria, gray = Deinococus-Thermus, and black = other bacterial phyla. Names of marine bacteria are colored blue and non-marine black. Midpoint rooting was used and likelihood values shown for each node. Scale bar represents changes per site. See Additional file 4 for detailed tree parameters. The NuoL homolog from *S. tropica *was used as an outgroup in the MrpD tree but is not shown.

**Figure 3:**
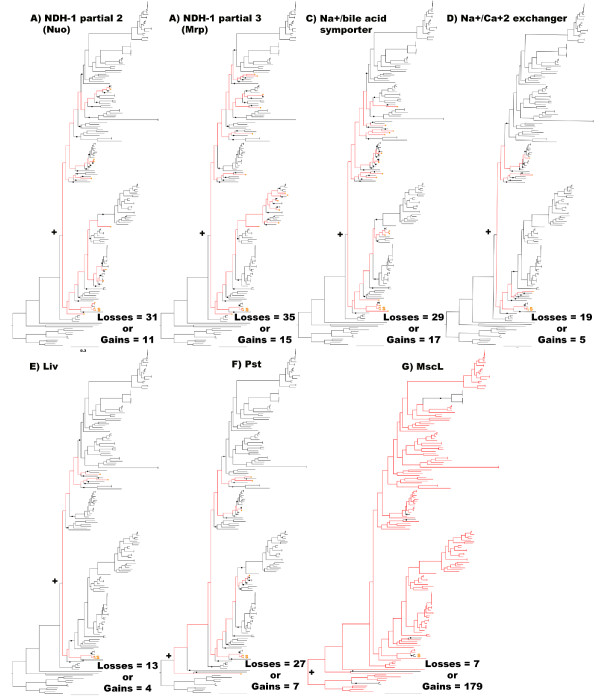
**Phylogenetic distributions of marine adaptation genes (MAGs) among the Actinobacteria**. Red branches in the species tree trace the occurrence of each MAG starting from the ancestor that accounts for all strains that maintain the gene (+). Black circles indicate the point in a lineage within which all strains lack the MAG. The minimum number of gene loss events was calculated by summing the black circles. The maximum number of gene gain events was calculated by summing the red circles. Pst, Liv, Partial 2, and Mrp represent all genes in the respective operons. S = *Salinispora*.

In contrast, phylogenetic analyses of all 13 genes in the second and third partial NDH-1 operons revealed close relationships with marine bacteria and thus these genes remained in the final MAG pool (Table 1). The annotation of the seven genes in the second partial NDH-1 operon predict that they encode the membranous portion of the enzyme complex, which pumps sodium ions or protons to generate an ionic motive force [30]. Among these seven genes, Stro769 and Sare711 are annotated as hypothetical proteins but likely encode NuoJ because top BLAST hits are annotated as such. The phylogenies of the corresponding seven Nuo protein sequences are similar and place them in a clade with nine other Actinobacteria (Figure 2A). The next three most closely related clades are comprised of nine Proteobacteria of which six are of marine origin. The Actinobacteria that possess these *nuo *genes are scattered throughout the species tree (Figure 3A), which could be interpreted as evidence for common ancestry within the Actinobacteria. To more formally infer the likelihood of gene loss (vertical inheritance) vs. gene gain (horizontal acquisition) in accounting for the distribution of these genes, the minimum number of loss events and maximum number of gain events was calculated. The resulting loss to gain ratio of 2.8 indicates that nearly three times as many loss events would be required to explain the observed distribution and thus provides support for the horizontal acquisition of this partial NDH-1 complex in *Salinispora *spp. (Figure 3A).

The six genes in the third partial NDH-1 complex have annotation related to *nuo *genes however upon closer analysis these genes appear to encode the sodium proton antiporter Mrp. The ambiguous annotation is not surprising as *mrp *genes are known to have sequence similarity to *nuo *genes [11]. Both *Salinispora *strains have *mrpA*-*G*, which are required for a functional antiporter [31], however *mrpA *and *B *are fused indicating that this is a group two Mrp operon [11]. *MrpG *was incorrectly predicted by auto-annotation but subsequently resolved based on homology with *B. halodurans*. MrpCEF and G are each too short to produce a robust phylogeny, however, the blast matches for these genes, and the fused *mrpAB *gene, were similar to the longer MrpD sequence and therefore it is inferred they share the same evolutionary history. The phylogeny of MrpD (Figure 2B) places the two *Salinispora *spp. in a primary clade that includes five *Corynebacteria *spp. and the Gram-negative marine bacterium *A. marinum*. This clade then shares a common ancestor with a large and diverse group of bacteria that contains at least four phyla including many marine and alkaliphilic species. The ratio of gene loss to gain events for each gene in the *mrp *operon is 2.3 (Figure 3B), thus supporting gene gain as the most parsimonious explanation for the occurrence of this gene in the two *Salinispora *spp.

**Figure 4:**
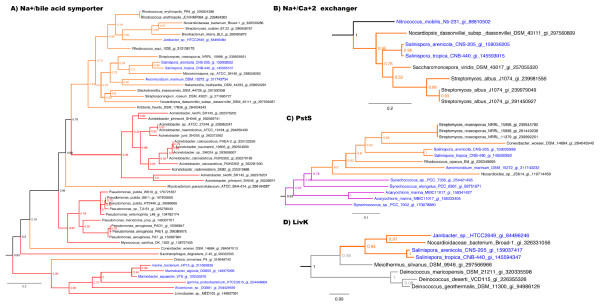
**Partial phylogenetic trees of four marine adaptation genes**. (A) Na^+^/bile acid symporter, (B) Na^+^/Ca^+2 ^exchanger, (C) PstS of the high affinity phosphate transporter, and (D) LivK from the branched chain amino acid transporter. Note: deep branches within the Actinobacteria are incongruent with the species phylogeny. Branches and species are colored as in Figure 2. See Additional file 4 for detailed tree parameters.

*S. tropica and S. arenicola *contain 18 and 19 candidate sodium transporter genes respectively (Table 1), three of which were confirmed as MAGs following phylogenetic analysis (Additional file 2). One of these MAGs constitutes a Na^+^/bile acid symporter (Stro2582 and Sare2779). The orthologs in the two *Salinispora *genomes group phylogenetically with 15 Actinobacteria including two other marine Actinobacteria (Figure 4A). The next clade contains *Acinetobacter *spp. followed by a single Actinobacterium and a large clade of *Pseudomonas *spp., many of which are human pathogens, and one *Myxococcus *sp. Subsequent clades include five Gram-negative marine bacteria. The apparent acquisition of this symporter may provide a mechanism to exploit a natural sodium gradient to import bile salts, which can be converted to compatible solutes such as glycine or taurine [32]. Interestingly, genes for the biosynthesis of the compatible solute glycine betaine were not found in either *Salinispora *genome while genes for the uptake of this compound displayed no evidence of acquisition from marine bacteria and thus were not identified as MAGs (data not shown). The second sodium transport gene is a Na^+^/Ca^+2 ^exchanger (Stro449 and Sare538) with phylogenetic links to three different Actinobacteria and then *Nitrococcus mobilis*, a member of the Gamma-proteobacteria derived from surface waters of the equatorial pacific (Figure 4B) followed by a group comprised entirely of marine proteobacteria (see treeBASE link provided in the Methods). The third gene is a Na^+^/Ca^+2 ^antiporter (Stro4216 and Sare4649) that is largely related to genes observed in marine Alpha-proteobacteria (data not shown). The gene loss to gain ratios of 1.7 and 3.8 for the Na^+^/bile acid symporter and Na^+^/Ca^+2 ^exchanger, respectively, supports the hypothesis that these genes were acquired. A gene loss to gain ratio was not calculated for the Na^+^/Ca^+2 ^antiporter because it was only observed in distantly related Actinobacteria and thus was assumed acquired. These calcium transporters may be related to the calcium requirement reported for *Salinispora *spp. [33].

TransportDB was used to identify 225 ABC transporters in each *Salinispora *genome (Additional file 2). After phylogenetic analysis of each protein, it was shown that the phosphate transporter Pst and branched chain amino acid transporter Liv have phylogenetic links to both marine and human associated bacteria (Figure 4C and 4D) and therefore advanced to the final MAG pool (Table 1). The four genes encoding the Pst transporter (Stro286-Stro289) display the same phylogenetic relationships and are closely related to homologs in marine cyanobacteria (Figure 4C). This transporter may be more efficient at scavenging phosphate from seawater than the form observed in soil Actinobacteria. The gene loss to gain ratio of 3.9 for each *pst *gene (Figure 3F) provides additional support for the acquisition of these genes in *Salinispora *spp. The five Liv proteins (Stro1801-1805) maintain the same phylogeny and reveal a close relationship to homologs from the marine Actinobacterium *Janibacter *sp. and then four bacteria from the Phylum Deinococcus-Thermus (Figure 4D). The next clade contains marine and pathogenic Proteobacteria (data not shown). The gene loss to gain ratio of 3.3 for each gene in this operon supports gene acquisition (Figure 3E).

Of the 35 and 33 channel and pore genes identified as candidate MAGs based on functional annotation in *S. tropica *and *S. arenicola*, respectively, 30 and 28 passed the phylogenetic test (Table 1). All of these were previously identified as polymorphic membrane proteins (Pmps) and showed a strong phylogenetic relationship with homologs in marine bacteria [25]. These genes are in high copy number (≥ 28) in both *Salinispora *genomes relative to the closely related genus *Micromonospora*, in which only two copies are observed. A structural alignment of the predicted Pmp proteins indicates that each forms a beta-barrel structure, which likely forms a pore in the membrane, and contains a signal sequence common to all Pmps supporting that these proteins target the cell membrane.

### Comparative genomics based identification of MAGs

A representative dataset comprised of 36 Actinobacterial genomes was used to identify 105 genes that are unique to both *Salinispora *spp. based on the RSD test of orthology (Table 1). Phylogenetic analyses revealed that seven of these genes shared a close relationship with homologs in marine bacteria and therefore advanced to the final MAG pool. However all seven of these genes were included among the MAGs previously identified based on gene function and thus comparative genomics revealed no new MAGS based on gene gain.

**Figure 5:**
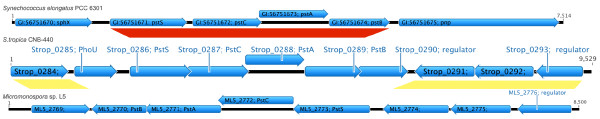
**Phosphate transport (*pst*) operon and surrounding region in *S. tropica *CNB-440**. Red box indicates synteny of *pst *between *S. tropica *CNB-440 and *Synechococcus elongatus *PCC6301. Yellow box indicates synteny between *S. tropica *CNB-440 and *Micromonospora *sp. L5. The GI numbers are listed for *S. elongatus *PCC6301, the locus tags are given for the *S. tropica *and *Micromonospora *genomes.

To assess gene loss based on comparative genomics, the *Micromonospora *sp. L5 genome was used as the reference sequence for the pair-wise RSD test of orthology in 27 representative Actinobacterial genomes, including both *Salinispora *spp. Four of 430 genes with predicted orthologs in at least 24 of the 27 genomes are absent in both *Salinispora *sequences (Additional file 2). These four genes are 1) a large conductance mechanosensitive channel (*mscL*) 2) an ABC transporter phosphate-binding protein (*pstS*), 3) a HAD-superfamily hydrolase, and 4) a peptidoglycan synthetase (*ftsI*). Homologs of *mscL *play a role in osmotic adaptation in halotolerant bacteria [34] and provide a mechanism to survive osmotic down shock [12,35]. Thus, the loss of this gene may play a key role in the inability of *Salinispora *strains to survive when transferred to low osmotic strength media. The gene loss to gain ratio for *mscL *is 0.04 and thus highly supports gene loss in both *Salinispora *spp. (Figure 3G). Based on the RSD analysis, *pstS *was also identified as being lost in both *Salinispora *spp. However, all four genes in the *pst *operon are present in both *Salinispora *genomes and were already identified as MAGs based on functional annotation and evidence they were acquired from marine cyanobacteria (Figure 4C). Thus, it appears that the *pst *genes observed in both *Salinispora *spp. were too divergent to be detected as orthologs based on a comparison with the *Micromonospora *L5 genome. In support of this, a synteny plot in the region of the *pst *operon suggests that a homologous recombination event has resulted in the replacement of the entire *Salinispora *operon with a cyanobacterial version (Figure 5). The HAD-superfamily hydrolase and peptidoglycan synthetase (*ftsI*) were not considered further as MAGs based on their functional annotation.

## Discussion

The marine Actinobacteria for which genome sequences are available are broadly distributed throughout the Actinobacterial phylogenetic tree and closely related to non-marine forms suggesting they have been independently introduced relatively recently into the marine environment. There is no evidence for a common set of genes linked to marine adaptation in these bacteria suggesting they have responded in different ways to the environmental pressures associated with survival in the marine environment. None of these bacteria, including the obligate marine genus *Salinispora*, possess Nqr, the sodium dependent respiratory NADH dehydrogenase that has frequently been linked to marine adaptation in Gram-negative marine bacteria [36]. Thus, there appear to be fundamental differences in the ways Gram-negative bacteria and the Gram-positive bacteria studied here have adapted to the marine environment.

Given that gene acquisition represents a major force driving bacterial evolution [37], it can be inferred that bacteria secondarily introduced into the marine environment will, over time, acquire adaptive traits from other marine bacteria. Using annotation as a guide, it was possible to identify a pool of genes in the two *Salinispora *genomes that are both relevant to marine adaptation and share a common evolutionary history with homologs from bacteria that inhabit hyper-osmotic environments. Despite the absence of Nqr, this pool includes 13 genes related to electron transport. These genes comprise two partial copies of NDH-1. One copy appears to encode the membranous portion of complex I, which pumps sodium ions or protons to generate an ionic motive force. The second copy contains *mrp *genes that likely encode a sodium antiporter that may help maintain a low cytoplasmic concentration of sodium. While Mrp is commonly found in bacteria and known to play a role in intracellular pH regulation [11], homologs in the two *Salinispora *spp. are distantly related to any previously described and may represent a new type of Mrp antiporter. Taken together, the two partial NDH-1 complexes likely give *Salinispora *spp. the ability to keep excess sodium out of the cytoplasm while helping to meet the challenges of maintaining a proton gradient in seawater, which typically has a pH of 8.3. None of the MAGs were related to the biosynthesis or acquisition of compatible solutes such as glycine betaine, and there was no evidence that any proteins have excessive amounts of acidic amino acids or hydrophobic residues (data not shown), suggesting they do not accumulate intracellular salts as a mechanism of osmoregulation.

Genome sequences for six Actinobacteria isolated from the marine environment were available at the time of this study. While the MAG pool identified in the two *Salinispora *genomes is not shared by any of these strains, the Na^+^/bile acid symporter is present in both *Janibacter *sp. and *A*. *marinum*. In addition, *A*. *marinum *also shares the MAGs *mrpD *and *pstS *with both *Salinispora *spp. while *livK *is also observed in *Janibacter *sp. The strain labeled 'marine actinobacterium' has none of the marine adaptation genes identified in the two *Salinispora *genome sequences. While all of the MAGs identified by gene gain were also identified by functional annotation, the *mscL *gene was uniquely identified as a MAG based on gene loss in *Salinispora *relative to other Actinobacteria. The loss of *mscL *is also observed in eight *Mobiluncus *species, *Streptomyces viridochromogenes*, *Streptomyces clavuligerus*, *Nocardiopsis dassonvillei*, *Rubrobacter xylanophilus*, and two *Collinsella *species and thus is not unique to *Salinispora *spp. These bacteria come either from sludge or a human source, two potentially consistent, hyper-osmotic environments where the loss of this gene may not prove disadvantageous. No other marine Actinobacteria have lost *mscL *and no Actinobacteria missing *mscL *have any of the *Salinispora *MAGs. These observations led to a series of genetic experiments that demonstrate the importance of MscL in allowing *Salinispora *strains to survive osmotic downshock (Bucarey et al., in prep.).

The phylogenies of all but one *Salinispora *MAG (Na^+^/Ca^+2 ^antiporter) contain non-marine Actinobacteria, which suggests these genes may also prove adaptive in other environments. For example, the human pathogen *Nocardiopsis dassonvillei *has three of the MAGs while *Brevibacterium linens*, *Streptomyces roseosporus*, *Streptosporangium roseum*, *Corynebacterium kroppensteddti*, *and Geodermatophilus obscurus *each possess two. In total MAG homologs were found in 32 non-marine Actinobacteria. As with the non-marine Actinobacteria that have lost *mscL*, many of these strains are human pathogens or were derived from activated sludge.

The key word searches and comparative genomics approaches used here yielded a pool of candidate MAGs that were subsequently tested for phylogenetic links to bacteria associated with hyperosmotic environments. The final list of MAGs is almost certainly incomplete, as the key word searches were limited and it is possible that adaptations to survival in marine sediments may be very different from those previously reported for seawater inhabiting bacteria. It is also possible that some genes involved in marine adaptation are widely distributed among Actinobacteria and thus would remain undetected using the comparative genomics approach. Likewise, gene mutation or duplication may lead to environmentally relevant adaptive traits that were not detected with the methods employed. While this study was not a comprehensive assessment of marine adaptation, it nonetheless identified a pool of acquired genes that appear to be highly relevant to the survival of *Salinispora *spp. in the marine environment.

## Conclusions

Functional annotation and comparative genomics were used to identify candidate marine adaptation genes in two *Salinispora *genome sequences. Using a phylogenomic approach, evidence of acquisition from bacteria associated with hyperosmotic environments was obtained for 57 and 59 genes in *S. arenicola *and *S. tropica*, respectively. An analysis of these genes reveals that the mechanisms of marine adaptation in *Salinispora *spp. are fundamentally different from those reported for Gram-negative bacteria and other marine Actinobacteria. While not comprehensive, the MAGs identified are largely associated with electron transport, sodium transporters, and ABC transporters and are predicted to represent marine adaptations based on evidence of acquisition from marine bacteria. The results also indicate that the loss of *mscL *may play a key role in the inability of *Salinispora *strains to survive osmotic down shock. Given that *Salinispora *spp. are a useful source of secondary metabolites with applications in human medicine [38], identifying the genetic basis for the osmotic requirements reported for this genus may prove useful for future industrial development.

## Methods

### Genome strains and analyses

The genomes of *S*. *tropica *strain CNB-440 (accession # CP000667) and *S*. *arenicola *strain CNS-205 (accession # CP000850) were downloaded from the U.S. Department of Energy Joint Genome Institute website (http://genome.ornl.gov/microbial/stro/03jan07 and http://genome.ornl.gov/microbial/sare/18jul07). Strains CNB-440 and CNS-205 were cultured from sediments collected at a depth of 20 m from the Bahamas and Palau, respectively. Artemis was used to visualize gene arrangement and annotation in each genome [39]. A Fasta file of predicted protein sequences from the two genomes served as a database for BLAST searches [40]. Candidate marine adaptation genes (MAGs) were identified based on 1) gene function (annotation-derived) and 2) comparative genomics. The resulting pool of candidate MAGs was then analyzed using phylogenetic approaches and those with evidence of a shared ancestry with bacteria associated with hyper-osmotic environments were kept in the final MAG pool. Thus, this study is largely focused on the identification of marine adaptation genes that were acquired from other marine bacteria.

### Function-based MAG identification

Keyword and BLAST searches were performed on the two *Salinispora *genomes using proteins previously linked to marine adaptation in studies of marine bacteria. The key words searched were associated with electron transport (complex I), sodium transporters, ABC transporters, and pores (Additional file 3). To improve the annotation of transporters prior to the key word searches, the two *Salinispora *genomes were submitted to transportDB [41], which annotates transporters according to the transport classification system [42]. The BLAST searches were performed using complex I genes or *mscL *(Additional file 3). All sequences identified using these methods were subject to phylogenetic analysis as described below.

### Comparative genomics-based MAG identification

Pair-wise comparisons were performed between *S. tropica *CNB-440 and 37 Actinobacterial genomes (including *S. arenicola *CNS-205) to identify orthologs that are present in both *Salinispora *genomes but absent in other Actinobacteria. The genomes selected for comparison include a broad phylogenetic range of Actinobacteria, three *Micromonospora *spp., and all marine Actinobacteria for which genomes sequences were available as of March 31, 2011. Orthologs were identified using the program Reciprocal Smallest Distance [43] based on e-values < 1e-5, no more than 50% sequence divergence over the entire alignment of the sequence, and the remainder of the parameters set at default. Orthologs were eliminated if they were < 350 amino acids in length or part of a mobile genetic element or secondary metabolite gene cluster as previously defined [25]. Orthologs that passed these criteria were then evaluated phylogenetically to determine if they had a shared evolutionary history with bacteria derived from hyper-osmotic environments.

The RSD test was also used to identify genes that were lost in the two *Salinispora *genomes relative to other Actinobacteria. In this case, the *Micromonospora *sp. L5 genome served as the reference for the pair-wise prediction of orthologs in 27 representative Actinobacterial genomes, including both *Salinispora *genomes. Sequences present in > 24 Actinobacterial genomes based on the above RSD criteria for orthology, but not in the two *Salinispora *genomes, were considered as candidates for gene loss. Functional annotation was then used to determine if gene loss could represent a marine adaptation.

### MAG phylogeny

All *Salinispora *protein sequences identified as candidate MAGs based on functional class and comparative genomics were subject to phylogenetic analysis to test for a shared evolutionary history with bacteria derived from hyper-osmotic environments. If a candidate MAG was part of an operon, the entire operon was tested. Maximum likelihood phylogenies were constructed for each candidate MAG using the online program MABL [44] with default settings (phylogeny.fr/version2_cgi/simple_phylogeny.cgi). The top 100 BLASTP hits were downloaded from the NCBI protein database and those with an e-value < 1e-5 and length greater than 50% of the alignment were included in the tree. Genes that claded with orthologs from hyper-osmotic environments and ≤ 25 Actinobacterial species were kept in the final MAG pool. In cases where the nearest clade was not entirely comprised of strains from hyper-osmotic environments but a majority of strains in all other major clades were, the gene was included in the final MAG pool. Exceptions included trees that contained two or more *Micromonospora *sequences, as this was viewed as evidence of vertical inheritance. The files used to create the trees shown in Figures 2 and 4 are available at http://purl.org/phylo/treebase/phylows/study/TB2:S12306.

### Species tree

All finished and several draft Actinobacterial genomes were downloaded from the NCBI FTP site on March 31, 2011. For Actinobacterial species with several draft genomes, at least two strains were included. In addition, any unnamed Actinobacterial species that contained a MAG were also included. The program AMPHORA [26] was then used to retrieve, align, and trim phylogenetic markers from each genome. Any marker that was not found in all species was excluded. If more than one version of a marker was found in a genome, the longest version that most closely fit the expected species phylogeny was selected. If the two versions were the same size and fit the expected phylogeny, one was selected randomly. Draft genomes were removed from the dataset if any marker gene was ≤ 25% of the size of all other sequences. However if the draft genome contained a MAG then none of the sequence data was removed. Finally, all aligned genes were concatenated and trimmed with Gblocks. The resulting alignment was input to PhyML for the construction of an Actinobacterial species tree.

### Quantification of gene gain and loss

The species tree was used to calculate whether horizontal gene transfer or vertical inheritance is the most parsimonious explanation for the observed evolutionary history of each MAG. This was done by first documenting the distribution of each MAG in the species tree. The minimum number of gene loss events was then calculated by identifying the deepest branches in the tree within which all strains lacked the MAG. These branches or points were then summed. The calculation started at the last common ancestor of all strains that possessed the gene. The maximum number of gene gain events was calculated assuming each MAG was acquired independently and summing the terminal branch tips representing each lineage in which the gene was observed. The ratio of the minimum number of gene loss events to the maximum number of gene gain events was then calculated and values above one considered to support the hypothesis that the gene was acquired (ie, a higher number of gene loss events would be required to account for the observed distribution and thus gene gain is the more likely explanation) while values below one were used to support gene loss.

## Authors' contributions

KP conceived the experimental design, performed data analyses, and drafted the manuscript. PRJ participated in the design and coordination of the study and prepared the final manuscript. Both authors read and approved the final manuscript.

## Supplementary Material

Additional file 1 **Genes used for species tree construction**. Click here for file

Additional file 2 **Complete list of candidate MAGs**. Click here for file

Additional file 3 **Keyword and BLAST query sequences**. Click here for file

Additional file 4 **Tree parameters and statistics generated by MABL**. Click here for file
